# Impulsivity, decision‐making, and risk behavior in bipolar disorder and major depression from bipolar multiplex families

**DOI:** 10.1002/brb3.3337

**Published:** 2023-12-18

**Authors:** Almudena Ramírez‐Martín, Lea Sirignano, Fabian Streit, Jerome C. Foo, Andreas J. Forstner, Josef Frank, Markus M. Nöthen, Jana Strohmaier, Stephanie H. Witt, Fermin Mayoral‐Cleries, Berta Moreno‐Küstner, Marcella Rietschel, Jose Guzmán‐Parra

**Affiliations:** ^1^ Department of Mental Health, University General Hospital of Malaga Biomedical Research Institute of Malaga (IBIMA) Malaga Spain; ^2^ Department of Genetic Epidemiology in Psychiatry, Central Institute of Mental Health, Medical Faculty Mannheim University of Heidelberg Mannheim Germany; ^3^ School of Medicine & University Hospital Bonn, Institute of Human Genetics, University of Bonn Bonn Germany; ^4^ Institute of Neuroscience and Medicine (INM‐1) Research Center Jülich Mannheim Germany; ^5^ Department of Personality, Assessment and Psychological Treatment University of Málaga Málaga Spain

**Keywords:** bipolar disorder, bipolar multiplex families, decision‐making, impulsivity, major depression disorder, risk behavior

## Abstract

**Objectives:**

Bipolar disorder (BD) and major depressive disorder (MDD) are characterized by specific alterations of mood. In both disorders, alterations in cognitive domains such as impulsivity, decision‐making, and risk‐taking have been reported. Identification of similarities and differences of these domains in BD and MDD could give further insight into their etiology. The present study assessed impulsivity, decision‐making, and risk‐taking behavior in BD and MDD patients from bipolar multiplex families.

**Methods:**

Eighty‐two participants (BD type I, *n* = 25; MDD, *n* = 26; healthy relatives (HR), *n* = 17; and healthy controls (HC), *n* = 14) underwent diagnostic interviews and selected tests of a cognitive battery assessing neurocognitive performance across multiple subdomains including impulsivity (response inhibition and delay aversion), decision‐making, and risk behavior. Generalized estimating equations (GEEs) were used to analyze whether the groups differed in the respective cognitive domains.

**Results:**

Participants with BD and MDD showed higher impulsivity levels compared to HC; this difference was more pronounced in BD participants. BD participants also showed lower inhibitory control than MDD participants. Overall, suboptimal decision‐making was associated with both mood disorders (BD and MDD). In risk‐taking behavior, no significant impairment was found in any group.

**Limitations:**

As sample size was limited, it is possible that differences between BD and MDD may have escaped detection due to lack of statistical power.

**Conclusions:**

Our findings show that alterations of cognitive domains—while present in both disorders—are differently associated with BD and MDD. This underscores the importance of assessing such domains in addition to mere diagnosis of mood disorders.

## 1 INTRODUCTION

Bipolar disorder (BD) and major depressive disorder (MDD) are highly prevalent (Hasin & Grant, 2015; Lia et al., 2012) and leading causes of disability worldwide (Jonas & Loeb, 2010). Although considerable insights in the etiology and pathophysiology of these disorders have been gained during recent years, there is still a fundamental lack of knowledge about their biological basis (Coleman et al., 2020), due in part to their biological and clinical complexity and heterogeneity (Merikangas et al., 2017). The diagnosis of a mood disorder is made categorically and based on the presence of a given number of clinical symptoms (out of a catalogue of possible symptoms), persisting over a defined period of time (World Health Organization; American Psychiatric Association, 2013). Thus, patients who receive the same diagnosis may differ with respect to symptoms, course of disease and disease etiology.

In order to improve disease characterization, research efforts have focused on defining core features of BD and MDD, one of which is alterations in neurocognitive function (Cotrena et al., 2020; Porter et al., 2015). Changes in neurocognitive functions such as cognitive impairment, especially in executive function, have been consistently reported in BD and MDD (Cotrena et al., 2020; Poletti et al., 2017). Executive dysfunction includes alterations in cognitive domains such as impulsivity, decision‐making, and risk‐taking behavior (Bora et al., 2013; Ramírez‐Martín et al., 2020). Changes in these domains have been assessed by self‐report and commonly used behavioral measures (Strickland & Johnson, 2021). Studies using such behavioral measures often distinguish two subtypes of impulsivity: (a) response inhibition assessed through Go/No‐Go tasks and (b) delay aversion assessed through gambling tasks (Ethridge et al., 2014; Powers et al., 2013). Gambling tasks, in which subjects are asked to choose between safe and risky alternatives in which quality of decisions can be assessed, are also used for the assessment of decision‐making and risk‐taking propensity (Strickland & Johnson, 2021).

These studies show that high levels of impulsivity, suboptimal decision‐making, and potentially dangerous risk behaviors are frequent components in the course of BD (Brambilla et al., 2013; Hıdıroğlu et al., 2013; Ramírez‐Martín et al., 2020) as well as in MDD (de Siqueira et al., 2018; Snyder, 2013). Both BD and MDD patients show alterations in impulsivity, decision‐making, and risk behavior compared to healthy participants (Alexander et al., 2017; Hart et al., 2019; Ozten et al., 2015; Sanches et al., 2014). Although impairment in impulsivity has been suggested as core feature of BD (Ramírez‐Martín et al., 2020), studies investigating the differences between BD and MDD have been sparse and do not report consistent findings (Porter et al., 2015). This may be due to the fact that prior studies have rarely carried out direct comparisons of these cognitive domains in BD and MDD patients using the same instruments.

The present study aimed to assess similarities and differences in impulsivity, decision‐making, and risk‐taking behavior in BD, MDD, and healthy controls. The present study was performed in BD and MDD members from bipolar multiplex families by the same raters (ARM and JGP).

## 2 MATERIALS AND METHODS

### 2.1 Participants

The sample consisted of participants from the Andalusian Bipolar Family (ABiF) study, a large study of multiplex bipolar disorder with comprehensive collection of phenotypic and biomaterial data. Family study methodology was employed, including direct interviews with affected individuals and first‐degree relatives by experienced psychiatrists and/or psychologists. Probands were recruited from index cases, both ambulatory and inpatient, and subsequently extended to other family members; for further details, see Guzman‐Parra et al. (2018). For the present study, data was used from a new recruitment wave with a focus on neuropsychological assessment and the collection of new biomaterial. The subsample for the present study included 82 participants: 25 BD type I, 26 MDD, 17 healthy relatives (HR), and 14 healthy controls (HC) who reside in Andalusia, Spain. BD, MDD, and HR participants were recruited from eight bipolar multiplex families, with a large share of the subjects stemming from two large families living in spatial proximity to the recruitment center University General Hospital of Malaga. The sample was comprised participants of Spanish descent, including male and female gender aged between 18 and 78 years; more detailed information about the individuals recruited from each family can be found in the Supplementary Material Table S1 and the pedigrees of the family 1 and 2 in the Supplementary Material Figures S5 and S6. HC were recruited from the general population. All participants provided written informed consent and the study was approved by the research ethics committee of the institution where assessment was performed.

### 2.2 Assessments

For the current subsample, a comprehensive neuropsychological assessment was conducted. The inclusion criteria for patients were: BD type I or MDD diagnosis and no manic symptoms at the time of assessment. HR had no personal history of lifetime BD or MDD, but as members of multiplex families did have a known family history of BD. Specifically, in this study, 11 HR had a first‐degree relative with BD and 6 had a first or second‐degree relative with MDD. HC had no personal history of lifetime BD or MDD, and no known history of BD in first‐degree relatives. More detailed sociodemographic and clinical information can be found in Table 1.

**TABLE 1:** Clinical and sociodemographic variables.

Variables	BD (*N* = 25)	MDD (*N* = 26)	HR (*N* = 17)	HC (*N* = 14)	*X* ^2^	*p*
Age: mean (*SD*)	52.84 (14.89)	52.58 (12.79)	52.24 (14.43)	43.64 (17.36)	3.407	.065
Gender (female); *n*, %	14 (56.00)	19 (73.08)	7 (41.18)	11 (78.57)	0.000	.995
Educational level^†^; *n*, %					6.802	.033
Primary	17 (68.00)	21 (80.77)	11 (64.71)	5 (35.71)		
Secondary	7 (28.00)	2 (7.69)	3 (17.65)	1 (7.14)		
University	1 (4.00)	3 (11.54)	3 (17.65)	8 (57.14)		
Age of onset: mean (*SD*)	20.88 (8.36)	36.46 (14.80)			25.860	3.664 × 10^−7^
CES‐D^‡^: mean (*SD*)	20.62 (14.75)	17.62 (13.04)	4.18 (5.10)	8.79 (8.87)	24.864	9.376 × 10^−7^

^†^ BD vs. HC: *X* ^2^ = 4.42, *p* = .035; MDD vs. HC: *X* ^2^ = 6.01, *p* = .014; BD vs. HR: *X* ^2^ = 25.98, *p* < .001; MDD vs. HR: *X* ^2^ = 51.14, *p* < .001.

^‡^ BD vs. HC: *X* ^2^ = 11.51, *p* = .001; MDD vs. C: *X* ^2^ = 36.80, *p* = .005.

BD = bipolar disorder; MDD = major depressive disorder; HR = healthy relatives; HC = healthy controls; *X* ^2^= chi‐square test; *p* = statistical significance; SD = standard deviation; *n* = number of cases; CES‐D = Center for Epidemiologic Studies Depression Scale.

### 2.3 Instruments

Data was collected using diagnostic interviews and neuropsychological assessment instruments. The diagnostic evaluation included the Structured Clinical Interview for DSM‐IV Research Version (SCID‐I) (First et al., 1994), the Operational Criteria Checklist for Psychotic Illness (OPCRIT) (McGuffin et al., 1991), a review of medical records, and interviews with first or second‐degree relatives using the Family Informant Schedule and Criteria (FISC) (Mannuzza et al., 1985). Current depressive symptoms were evaluated with the Center for Epidemiologic Studies Depression Scale (CES‐D) (Radloff, 1977).

Impulsivity, decision‐making, and risk behavior were evaluated using computer‐based neuropsychological tasks. These instruments evaluate the different cognitive domains from a behavioral paradigm. Selected tests (specified below) of the Cambridge Neuropsychological Test Automated Battery (CANTAB) (Sahakian & Owen, 1992) were used for neuropsychological evaluation. More information about CANTAB can be found elsewhere (https://www.cambridgecognition.com/cantab).

#### 2.3.1 Impulsivity

Behavioral measures of impulsivity were subdivided into those exploring (1) response inhibition and (2) delay aversion, that is, the sensitivity to delayed consequences.

Response inhibition was evaluated using the Stop Signal Task (SST). This instrument instructs participants to respond as quickly as possible to a stimulus (an arrow pointing left or right) using a cursor with two buttons (go‐trial) and not to respond when an acoustic signal appears (stop‐trial; 25% of trials). The time between the appearance of the arrow and the acoustic signal in the stop‐trials is adapted to the performance of the participant starting at 250 milliseconds (ms), if participant`s response is correct it decreases 50 ms, and if it is incorrect it increases 50 ms. The main measure selected was “the stop‐signal reaction time” (SSRT), which is defined as the average reaction time necessary for the participant to correctly inhibit response on 50% of stop‐trials. Therefore, higher reaction times indicate more impaired response inhibition.

Delay aversion was evaluated using the Cambridge Gambling Task (CGT). The task consists of the presentation of ten squares at the top of the screen, some of which are red and others blue, with the proportions of colors different in each presentation. Under one of the squares, a yellow token is hidden and the participant has to try to find out if it is under a blue or red square. The participants start with 100 points and can select a proportion of points to bet on their decision. If the participant correctly selects under which color the yellow token is, they will accumulate the amount of points selected; if not, this number of points will be taken away. The current bet value is displayed in a box on the right of the screen and will either start low and increase (ascending condition) or start high and decrease (descending condition). The participant is instructed to try to earn as many points as possible without running out of points. The main measure of assessment selected was “delay aversion,” which is operationalized as the difference in average betting ratios chosen across ascending and descending conditions, but only counting the optimal trials (i.e., where the participant chose the color that had the greater number of boxes) expressed in percentage. Large differences between conditions correspond to more rapid, impulsive betting.

#### 2.3.2 Decision‐making

Decision‐making was evaluated using the “quality of the decisions made” (QDM) on the CGT. This outcome measure is the percentage of trials where a participant has chosen the color with more boxes. Higher percentage indicates better quality of decisions.

#### 2.3.3 Risk behavior

Risk behavior was evaluated using the “risk‐taking” outcome measure from the CGT. The outcome is the mean percentage of the total points that participants decide to gamble, on trials with the most likely outcome selected. A high score indicates decreased risk‐taking.

**FIGURE 1:**
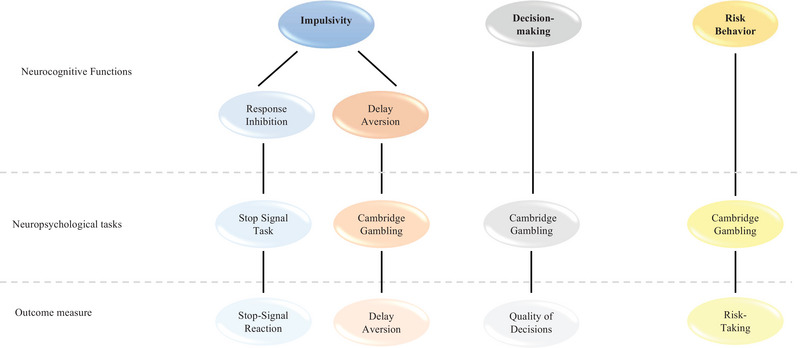
Neuropsychological tasks used to assess different cognitive domains from a behavioral paradigm.

A summary of the tasks used and the different cognitive domains evaluated are shown in Figure 1.

### 2.4 Statistical analysis

We used generalized estimating equations (GEEs) for association testing, a method that can deal with the peculiarities of the correlation structure posed by family data and which we already had applied successfully earlier (Guzman‐Parra et al., 2021). With this approach we tested whether diagnostic groups—namely healthy controls, healthy relatives, participants with unipolar depression and participants with bipolar disorder differed from each other in response inhibition, delay aversion, decision‐making, and risk behavior. Underlying correlation structure for GEEs was specified as exchangeable, which is suitable for data with family structure (Ziegler et al., 1998). The association of groups with CES‐D score, educational level, age, and sex were tested; significant differences between the groups were found for CES‐D score and educational level and were therefore used as covariates in all analyses. As a sensitivity analysis, age and sex were added to the GEE analysis. As an additional sensitivity analysis, with the aim to assess the robustness of the GEE models, an alternative random effects linear modeling approach was carried out, including clustered errors robust against heterogeneity of variances, with errors clustered according to the family structure. Deviating results of the sensitivity analyses are noted in the results section, and full results can be found in Supplementary Material Tables S2–S4. For all analyses, the alpha level was set to .05. SPSS version 25 (SPSS Inc., USA) was used for all analyses except for the linear mixed modeling approach in sensitivity analyses, where R v4.1.3 with package plm v2.6‐2 (https://.r‐project.org/) was applied.

## 3 RESULTS

**FIGURE 2:**
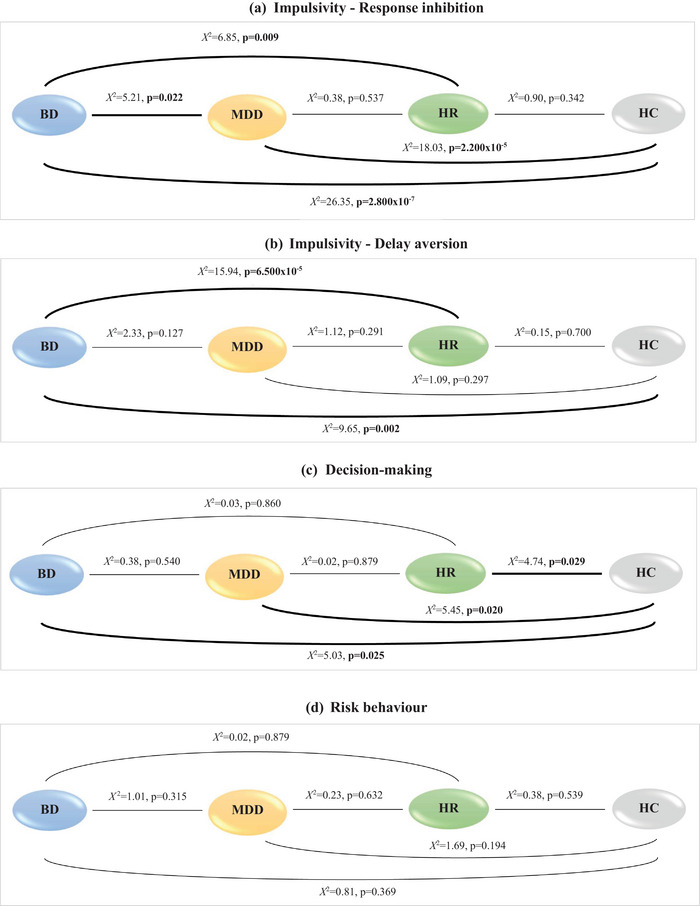
**(a–d)** Graphical representation of the results between‐group comparison on response inhibition, delay aversion, decision‐making and risk behavior. Note: The bold line indicates the significant differences between groups. BD = bipolar disorder; MDD = major depressive disorder; HR = healthy relatives; HC = healthy controls; *X* ^2^= chi‐square test; *p* = statistical significance.

**FIGURE 3:**
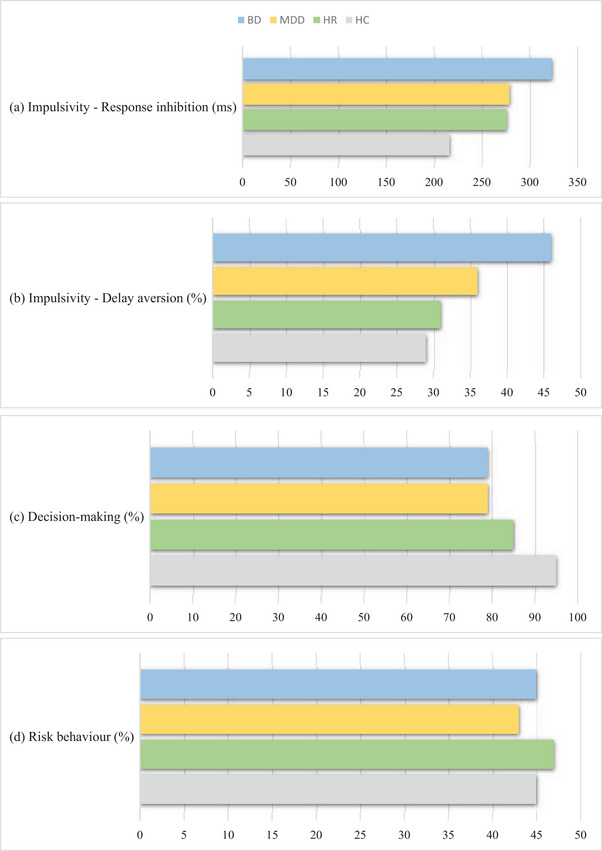
Bar chart of task outcome for each group. BD = bipolar disorder; MDD = major depressive disorder; HR = healthy relatives; HC = healthy controls.

Results are presented for each of the cognitive domains in Figures 2a–d and 3. Tables 2 and 3 show the results in different groups and between‐group comparison of response inhibition, delay aversion, decision‐making, and risk behavior.

**TABLE 2:** Response inhibition, delay aversion, decision‐making, and risk behavior in the different groups.

	BD, *N* = 25 *M* (*SD*)	MDD, *N* = 26 *M* (*SD*)	HR, *N* = 17 *M* (*SD*)	HC, *N* = 14 *M* (*SD*)
Stop Signal Task (response inhibition)				
SSRT (millisecond)	323.25 (108.57)	278.82 (88.62)	276.09 (132.98)	216.30 (42.10)
Cambridge gamble task (delay aversion, decision‐making, and risk behavior)				
Delay aversion (%)	46 (0.22)	36 (0.24)	31 (0.22)	29 (0.19)
Quality of decision (%)	79 (0.20)	79 (0.21)	85 (0.19)	95 (0.05)
Risk‐taking (%)	45 (0.12)	43 (0.16)	47 (0.14)	45 (0.14)

BD = bipolar disorder; MDD = major depressive disorder; HR = healthy relatives, HC = healthy controls; *M* = mean; *SD* = standard deviation; SSRT = stop‐signal reaction time.

**TABLE 3:** Between‐group comparison of response inhibition, delay aversion, decision‐making, and risk behavior.

	BD vs. MDD *X* ^2^, *p*	BD vs. HR *X* ^2^, *p*	BD vs. HC *X* ^2^, *p*	MDD vs. HR *X* ^2^ *, p*	MDD vs. HC *X* ^2^, *p*	HR vs. HC *X* ^2^, *p*	*X* ^2^, *p*
Stop Signal Task (response inhibition)							
SSRT	5.21, 0.022	6.85, 0.009	26.35, 2.823 × 10^−7^	0.38, 0.537	18.03, 2.200 × 10^−5^	0.90, 0.342	50.56, < 6.076 × 10^−11^
Cambridge gamble task (delay aversion, decision‐making, and risk behavior)							
Delay aversion	2.33, 0.127	15.94, 6.500 × 10^−5^	9.65, 0.002	1.12, 0.291	1.09, 0.297	0.15, 0.700	18.75, 3.090 × 10^−4^
Quality of decision	0.38, 0.540	0.03, 0.860	5.03, 0.025	0.02, 0.879	5.45, 0.020	4.74, 0.029	12.90, 0.005
Risk‐taking	1.01, 0.315	0.02, 0.879	0.81, 0.369	0.23, 0.632	1.69, 0.194	0.38, 0.539	2.38, 0.497

BD = bipolar disorder; MDD = major depressive disorder; HR = healthy relatives; HC = healthy controls; *X* ^2^= chi‐square test; *p* = statistical significance; SSRT = stop‐signal reaction time.

### 3.1 Impulsivity

#### 3.1.1 Response inhibition

BD had significantly longer SSRT than MDD participants (BD: 323.25 ms vs. MDD: 278.82 ms, *X*2= 5.21, *p* = .022). While BD had longer SSRT than HR (BD: 323.25 ms vs. HR: 276.09 ms, *X* ^2^ = 6.85, *p* = .009), there was no significant difference between MDD and HR (MDD: 278.82 ms vs. HR: 276.09 ms, *X* ^2^ = 0.38, *p* = .537). Both BD and MDD had significantly longer SSRT than HC (BD: 323.25 ms vs. HC: 216.30 ms, *X* ^2^ = 26.35, *p* = 2.823 × $10^{-7}$; MDD: 278.82 ms vs. HC: 216.30 ms, *X* ^2^ = 18.03, *p* = 2.200 ×$\;10^{-5}$). There were no significant differences in SSRT between HR and HC (HR: 276.09 ms vs. HC: 216.30 ms, *X* ^2^ = 0.90, *p* = .342) (see Figure 2a).

#### 3.1.2 Delay aversion

BD and MDD did not differ significantly in their CGT scores for delay aversion (BD: 46% vs. MDD: 36%, *X* ^2^ = 2.33, *p* = .127). However, BD showed significantly higher scores compared to HR (BD: 46% vs. HR: 31%, *X* ^2^ = 15.94, *p* = 6.500 ×$\;10^{-5}$) but MDD and HR did not differ significantly (MDD: 36% vs. HR: 31%, *X* ^2^ = 1.12, *p* = .291). In line with this, BD also presented with significantly higher scores compared to HC (BD: 46% vs. HC: 29%, *X* ^2^ = 9.65, *p* = .002), whereas MDD had no significantly higher scores than HC (MDD: 36% vs. HC: 29%, *X* ^2^ = 1.09, *p* = .297). Lastly, HR and HC showed no significant differences in their scores (HR: 31% vs. HC: 29%, *X* ^2^ = 0.15, *p* = .700) (see Figure 2b).

### 3.2 Decision‐making

BD and MDD groups showed no significant differences in their CGT scores for quality of decision‐making (BD: 79% vs. MDD: 79%, *X* ^2^ = 0.38, *p* = .540). No significant differences were observed between BD and HR (BD: 79% vs. HR: 85%, *X* ^2^ = 0.03, *p* = .860) and between MDD and HR (MDD: 79% vs. HR: 85%, *X* ^2^ = 0.02, *p* = .879). In contrast, significant differences were found between BD and HC (BD: 79% vs. HC: 95%, *X* ^2^ = 5.03, *p* = .025), MDD and HC (MDD: 79% vs. HC: 95%, *X* ^2^ = 5.45, *p* = .020), and also when comparing HR and HC (HR: 85% vs. HC: 95%, *X* ^2^ = 4.74, *p* = .029) (see Figure 2c).

### 3.3 Risk behavior

In risk behavior, there were no significant differences in CGT scores for risk‐taking between BD and MDD groups (BD: 45% vs. MDD: 43%, *X* ^2^ = 1.01, *p* = .315). Taking the comparisons with HR, no significant differences were found between groups (BD: 45% vs. HR: 47%, *X* ^2^ = 0.02, *p* = .879; MDD: 43% vs. HR: 47%, *X* ^2^ = 0.23, *p* = .632). Likewise, there were no significant differences between BD and HC (BD: 45% vs. HC: 45%, *X* ^2^ = 0.81, *p* = .369) and between MDD and HC (MDD: 43% vs. HC: 45%, *X* ^2^ = 1.69, *p* = .194). Finally, comparing HR and HC there were no significant differences in risk‐taking scores (HR: 47% vs. HC: 45%, *X* ^2^ = 0.38, *p* = .539) (see Figure 2d).

### 3.4 Sensitivity analyses

The sensitivity analyses (GEE models additionally adjusting for age and sex; linear models with random effects) revealed largely consistent results (for full results, see Supplementary Material Tables S2–S4). In the alternative linear models, the difference HR < HC in decision‐making (GEE: *p* = .029; linear model: *p* = .077); and in the GEE models additionally adjusting for sex and age, for response inhibition the difference between BD and HR (GEE: *p* = .009; GEE adjusting for age and sex: *p* = .069), and for decision‐making, the differences between BD and HC (GEE: *p* = .025; GEE adjusting for age and sex: *p* = .071) and HR and HC (GEE: *p* = .029; GEE adjusting for age and sex: *p* = .056) were less significant. Also, to quantify the influence of the largest families on the results, we report the raw values of the different subsamples (total sample, families 1 and 2 separately, all families excluding family 1 and family 2) in the Supplementary Figures S1–S4.

## 4 DISCUSSION

This is the first study that compares BD, MDD, HR, and HC with respect to impulsivity, decision‐making, and risk behavior, conducted in high‐density bipolar families. Higher impulsivity was observed in participants with BD and MDD when compared to HC, with lower response inhibition in BD when compared with MDD participants. Lower decision‐making was associated with mood disorders but did not differ between BD or MDD. Finally, no differences in risk‐taking behavior were found between groups.

### 4.1 Impulsivity

Our results support prior findings showing a strong link between impulsivity and BD (Bauer et al., 2017; Bora et al., 2009; Ramírez‐Martín et al., 2020; Strakowski et al., 2010), with impairments on response inhibition and delay aversion in BD in comparison to controls. The present results are consistent with other studies, which have found more impairment in inhibitory control in BD compared with MDD (Cotrena et al., 2016). Furthermore, other studies support our finding that also MDD patients have impaired response inhibition (Aker et al., 2016; Li et al., 2021). In contrast, some studies have not reported an association between impulsivity and mood disorders (Huang et al., 2022; Newman & Meyer, 2014). Regarding this, it is important to point out that the evidence reported was different between studies employing measures of trait impulsivity (in self‐report format) and studies exploring impulsivity with behavioral paradigms (Newman & Meyer, 2014). Most studies using self‐report measures reported significant differences between BD and MDD patients and healthy controls (Ozten et al., 2015; Saddichha & Schuetz, 2014), whereas when measured by behavioral paradigms the evidence is contradictory. This may be due to the fact that studies that used self‐reports unanimously used The Barratt Impulsiveness Scale (BIS‐11) (Patton et al., 1995), and studies that assessed impulsivity from a behavioral paradigm rarely carried out the assessment using the same instruments. Our results, therefore, support this body of research by extending similar findings to behavioral measures of impulsivity. Differences between BD and healthy individuals (HR and HC), but not between MDD and HR suggest that impulsivity is a trait that is stronger in BD than in MDD. It should be noted that the MDD patients from multiplex families examined here have a high genetic burden for BD, and it might be expected that MDD patients from those families would show more impulsivity than MDD patients from the general population. In light of this, it is notable that the BD patients show a significantly stronger impairment of response inhibition compared to MDD patients from these high‐density families, suggesting that inhibitory control is an important cognitive feature when differentiating mood disorder subtypes.

### 4.2 Decision‐making

No differences were found between BD and MDD with respect to decision‐making. This is in line with the prior suggestion that impairment of decision‐making is a transdiagnostic feature of MDD and BD (Richard‐Devantoy et al., 2016) and as well of SCZ (Ting et al., 2020). The observation that quality of decisions in HR participants did not differ from BD and MDD but from HC suggests that a high genetic burden for affective disorders might affect decision‐making even without a clinical diagnosis of BD or MDD.

### 4.3 Risk behavior

The absence of group differences in risk behavior in our results comes in contrast to previous research reporting differences in risk behavior between BD and HC (Hart et al., 2019; Hıdıroğlu et al., 2013). This could be due to the limited sample size of our study, or on the other hand, it may be that group differences in risk behavior are negligible. The latter would be in line with a recent meta‐analysis, which also did not find a significant difference in risk behavior between BD and HC (Ramírez‐Martín et al., 2020).

### 4.4 Limitations and strengths

The present findings must be interpreted in light of some limitations. One limitation is that study groups were not counterbalanced across demographic characteristics, such as age, gender, and education, although it has to be mentioned that participants did not differ significantly in their sociodemographic variables (see Table 1). Variables with significant differences between groups (current depression and educational level) were adjusted for in our models. In sensitivity analyses, the models were additionally adjusted for age and sex, with largely consistent results. As GEE models in smaller samples might be biased, we replicated the results using a linear mixed modeling approach, again with largely similar results. However, the limitation of the small sample size might have made it difficult to detect small differences between multiple groups. Replicating the present results in larger samples will allow for a more reliable estimation of the associations. Another shortcoming is that the used CANTAB battery focused on specific modules, limiting its generalizability across different cognitive domains. Also, from the CANTAB test battery, no general cognition factor can be derived which would have been a better proxy for intelligence than education.

The main strength of this study is the standardized assessment and comparison of BD and MDD patients stemming from multiplex families. Both the standardized assessment by the same raters and the common genetic descent (familial background) reduce heterogeneity, which otherwise may blur results, especially when sample size is limited. On the other hand, if differences are found they are likely to be true differences. Samples like this are rare and a precious resource to discover important features of mood disorder subtypes.

In conclusion, the present investigation of multiplex families with a high genetic load for BD contributes to the current knowledge with respect to neurocognitive functioning in mood disorders. We found that impairment in response inhibition is strongly related to BD diagnosis, underlining the importance of inhibitory control as a cognitive feature for the characterization of mood disorder subtypes. Furthermore, our finding that MDD also has impairment in impulsivity, albeit less than BD, adds to the discussion whether MDD and BD should still be considered as distinct entities. Findings from this study in bipolar multiplex families are congruent with the trend toward a dimensional characterization of mood disorders rather than simple categorical diagnostic concepts (Morris & Cuthbert, 2012). Further studies with larger samples are needed to clarify the complex architecture of mood disorders and their association with impulsivity, decision‐making, and risk behavior.

## AUTHOR CONTRIBUTIONS

**Almudena Ramírez‐Martín and Jerome C. Foo**: Conceptualization; methodology; writing—review and editing. **Lea Sirignano and Fabian Streit**: Conceptualization; funding acquisition; methodology; writing—review and editing. **Andreas J. Forstner; Markus M. Nöthen; Jana Strohmaier and Stephanie H. Witt**: Conceptualization; writing—review and editing. **Josef Frank**: Formal analysis; writing—review and editing. **Fermin Mayoral‐Cleries and Berta Moreno-Küstner**: Investigation; resources. **Marcella Rietschel**: Conceptualization; funding acquisition; investigation; methodology; resources; supervision; writing—review and editing. **Jose Guzmán‐Parra**: Conceptualization; formal analysis; investigation; methodology; resources; supervision; writing—review and editing.

## CONFLICT OF INTEREST STATEMENT

All authors declare that they have no conflict of interest.

### PEER REVIEW

The peer review history for this article is available at https://publons.com/publon/10.1002/brb3.3337.

## Supporting information

Table S1. Information on individuals recruited from each family. Total number of pedigrees and number of individuals per pedigree.
Table S2. Between‐group comparison of response inhibition, delay aversion, decision‐making, and risk behavior in alternative approach.
Table S3. Between‐group comparison of response inhibition, delay aversion, decision‐making, and risk behavior in alternative approach with sex and age adjustment.
Table S4. Between‐group comparison of response inhibition, delay aversion, decision‐making, and risk behavior with GEE models additionally adjusting for age and sex.
Figure S1. Stop Signal Task comparison of subgroups.
Figure S2. Delay aversion comparison of subgroups.
Figure S3. Quality of decision‐making comparison of subgroups.
Figure S4. Risk‐taking comparison of subgroups.
Figure S5. Pedigree of family 1, including 24 participants. Family 1 was divided in subpedigrees 1–8.
Figure S6. Pedigree of family 2, including 23 participants. Family 2 was divided in 8 subpedigrees.

## Data Availability

The data are available upon reasonable request to the principal investigators of the ABiF project.
