# Supplementary material for: Impulsivity, decision‐making, and risk behavior in bipolar disorder and major depression from bipolar multiplex families
Source: Brain Behav. 2023 Dec 18;14(2):e3337. doi: 10.1002/brb3.3337 (PMC10897498; doi:10.1002/brb3.3337)

**Supplementary Material**

**Table S1.** Information on individuals recruited from each family. Total number of pedigrees and number of individuals per pedigree.

| Number of family | Total number of individuals | No. of BD-I individuals | No. of MDD individuals | No. of relatives individuals |
| --- | --- | --- | --- | --- |
| Family 1 | 24 | 10 | 8 | 6 |
| Family 2 | 23 | 5 | 10 | 8 |
| Family 3 | 3 | 1 | 2 | 0 |
| Family 4 | 4 | 2 | 2 | 0 |
| Family 5 | 3 | 2 | 1 | 0 |
| Family 6 | 3 | 1 | 0 | 2 |
| Family 7 | 6 | 3 | 3 | 0 |
| Family 8 | 2 | 1 | 0 | 1 |

**Table S2.** Between-group comparison of response inhibition, delay aversion, decision-making and risk behavior in alternative approach.

|  | BD vs. MDD  *p* | BD vs. HR  *p* | BD vs. HC  *p* | MDD vs. HR  *p* | MDD vs. HC  *p* | HR vs. HC  *p* |
| --- | --- | --- | --- | --- | --- | --- |
| Stop Signal Task (Response inhibition)  SSRT | 0.017 | 0.010 | 2.9x10^-6^ | 0.63 | 0.00027 | 0.23 |
| Cambridge gamble task (Delay aversion, decision-making and risk behaviour)  Delay aversion  Quality of decision  Risk-taking | 0.088  0.78  0.35 | 3.3x10^-5^  0.87  0.89 | 0.0022  0.040  0.40 | 0.30  0.75  0.65 | 0.20  0.033  0.22 | 0.79  0.077  0.56 |

BD = Bipolar Disorder; MDD = Major Depressive Disorder; HR = Healthy relatives; HC = Healthy controls; *p* = statistical significance; SSRT = stop-signal reaction time.

**Table S3.** Between-group comparison of response inhibition, delay aversion, decision-making and risk behavior in alternative approach with sex and age adjustment.

|  | BD vs. MDD  *p* | BD vs. HR  *p* | BD vs. HC  *p* | MDD vs. HR  *p* | MDD vs. HC  *p* | HR vs. HC  *p* |
| --- | --- | --- | --- | --- | --- | --- |
| Stop Signal Task (Response inhibition)  SSRT | 0.014 | 0.089 | 9.6x10^-7^ | 0.53 | 0.0033 | 0.48 |
| Cambridge gamble task (Delay aversion, decision-making and risk behaviour)  Delay aversion  Quality of decision  Risk-taking | 0.12  0.76  0.57 | 2.3x10^-5^  0.92  0.90 | 0.0035  0.11  0.51 | 0.31  0.79  0.76 | 0.22  0.06  0.33 | 0.90  0.12  0.68 |

BD = Bipolar Disorder; MDD = Major Depressive Disorder; HR = Healthy relatives; HC = Healthy controls; *p* = statistical significance; SSRT = stop-signal reaction time.

**Table S4.** Between-group comparison of response inhibition, delay aversion, decision-making and risk behavior with GEE models additionally adjusting for age and sex.

|  | BD vs. MDD  *X^2^, p* | BD vs. HR  *X^2^, p* | BD vs. HC  *X^2^, p* | MDD vs. HR  *X^2^, p* | MDD vs. HC  *X^2^, p* | HR vs. HC  *X^2^, p* |
| --- | --- | --- | --- | --- | --- | --- |
| Stop Signal Task (Response inhibition)  SSRT | 7.10, 0.008 | 3.30, 0.069 | 30.78, 2.886x10^-8^ | 0.45, 0.501 | 10.49, 0.001 | 0.66, 0.416 |
| Cambridge gamble task (Delay aversion, decision-making and risk behaviour)  Delay aversion  Quality of decision  Risk-taking | 2.81, 0.094  0.09, 0.767  0.426, 0.514 | 17.94, 2.300x10^-5^  0.01, 0.905  0.02, 0.897 | 8.88, 0.003  3.26, 0.071  1.06, 0.302 | 1.16, 0.281  0.08, 0.775  0.11, 0.740 | 1.73, 1.88  4.42, 0.036  1.85, 0.174 | 0.25, 0.873  3.67, 0.056  0.53, 0.469 |

BD = Bipolar Disorder; MDD = Major Depressive Disorder; HR = Healthy relatives; HC = Healthy controls; *X^2^* = Chi-square test; *p* = statistical significance; SSRT = stop-signal reaction time.

**Supplementary Figures**

Boxplots of raw measurement values of the groups. In addition, single values of every person are inset as gray dots. Group names: HC: healthy controls, HR: healthy relatives, MDD: major depressive disorder patients, BD: bipolar disorder patients; in subpanels C and D HCs are shown for comparison reasons only and are not actually members of the respective families, therefore the boxplot is transparent.

**Figure S1.** Stop Signal Task Comparison of Subgroups.


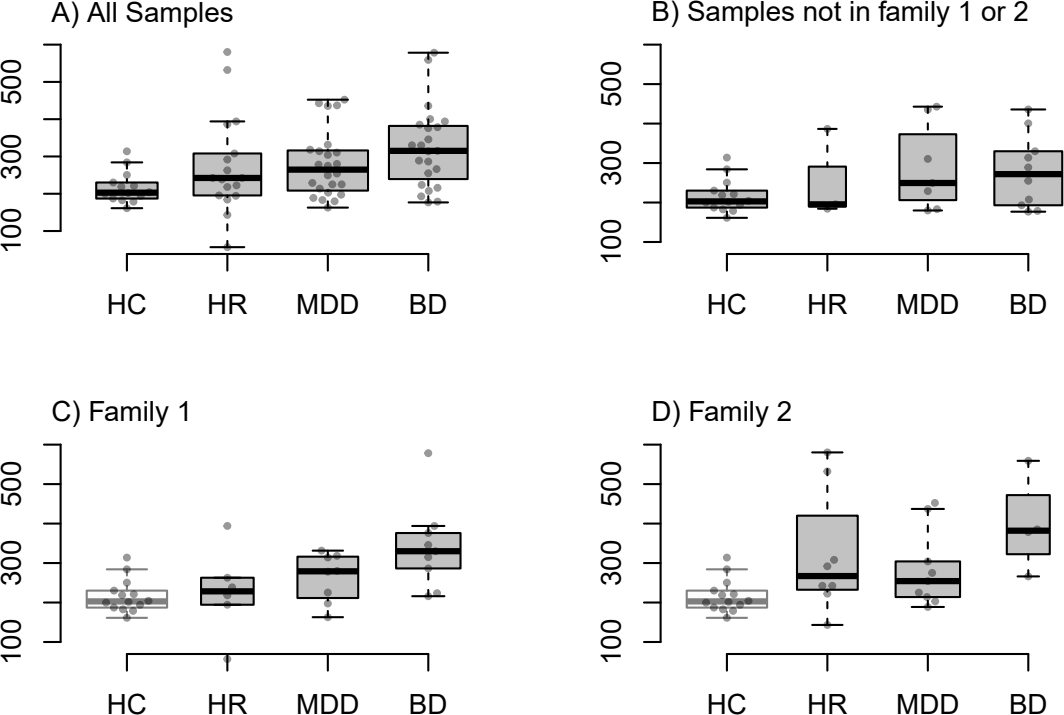


**Figure S2**. Delay Aversion Comparison of Subgroups.


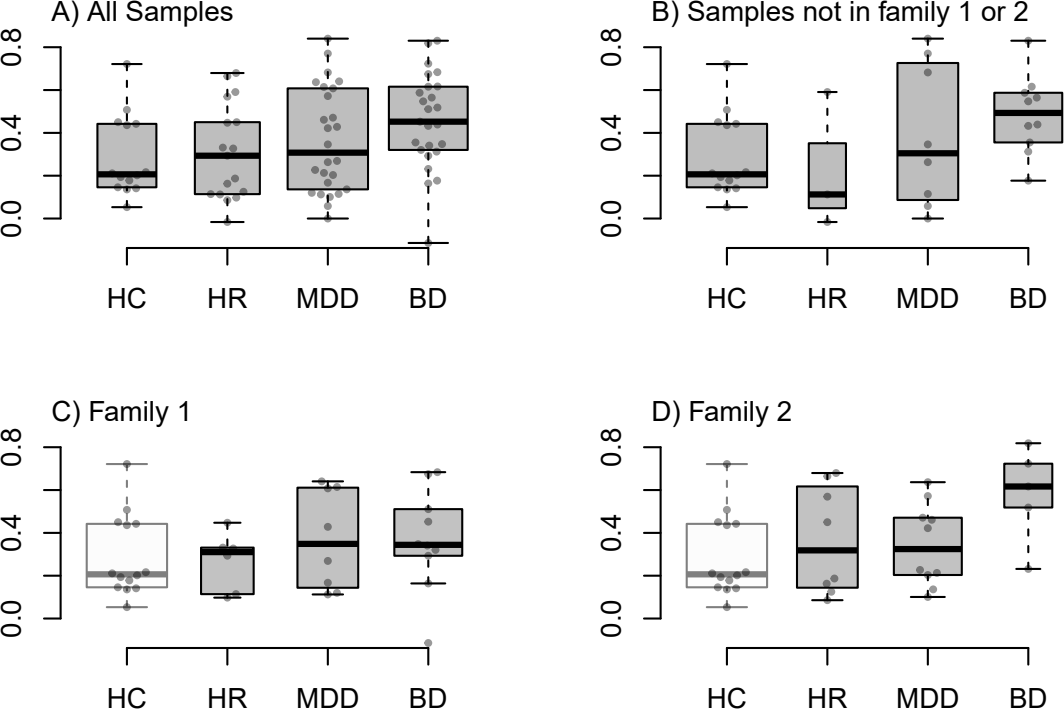


**Figure S3**. Quality of decision-making Comparison of Subgroups .


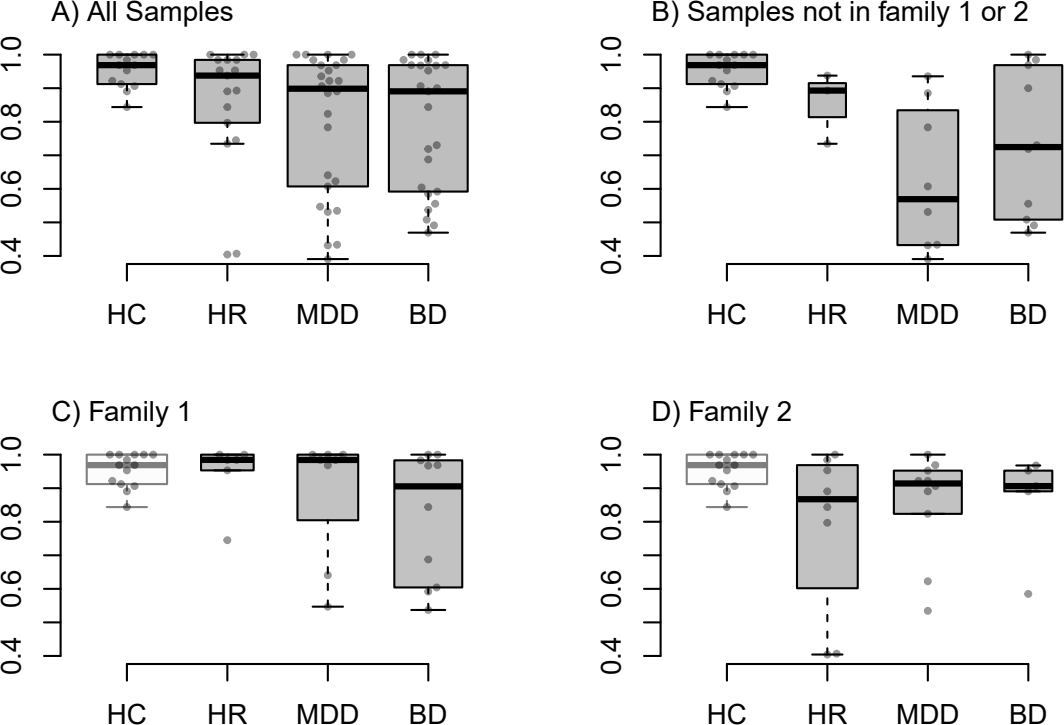


**Figure S4**. Risk-taking Comparison of Subgroups.


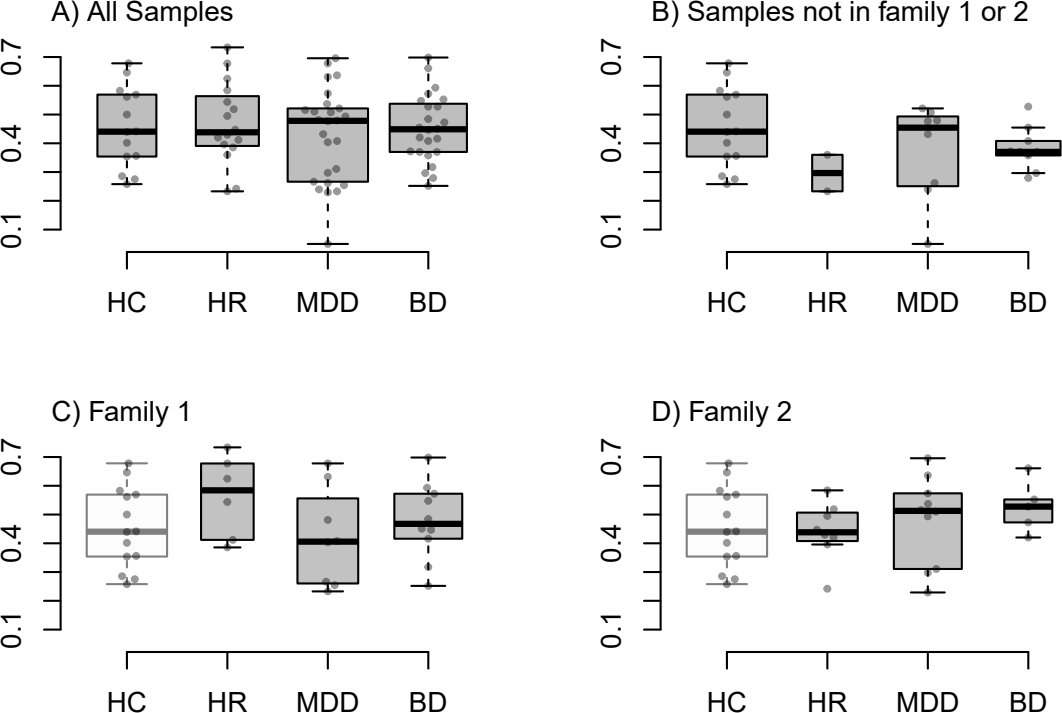


Pedigrees of the two large families. To preserve the anonymity and confidentiality of the families, no information is shown concerning gender or age of participants and the interrelations between the subpedigrees that make up the family. Individuals with first-degree relatives with BD are represented by a diamond with a central dot. Subjects included in the study are framed with a red circle. Blue indicates a diagnosis of BD type I. Yellow indicates a diagnosis of MDD. Unaffected individuals are indicated by white diamonds.

**Figure S5**. Pedigree of family 1, including twenty-four participants. Family 1 was divided in subpedigrees 1 to 8.


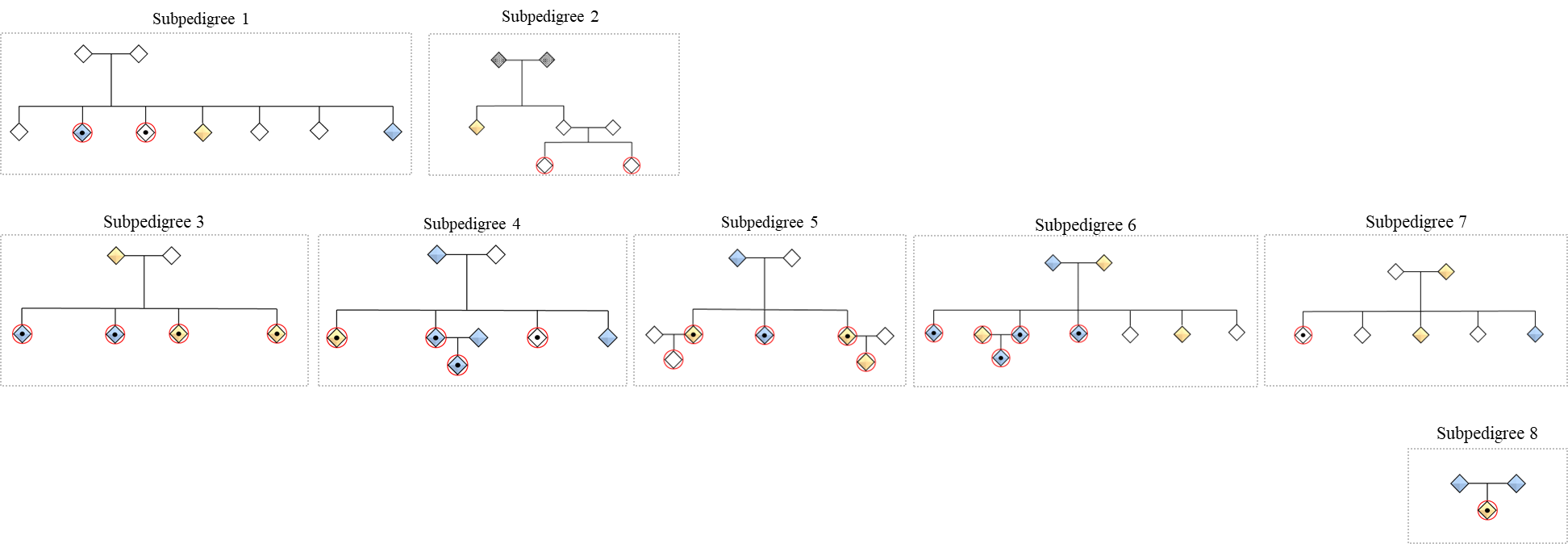

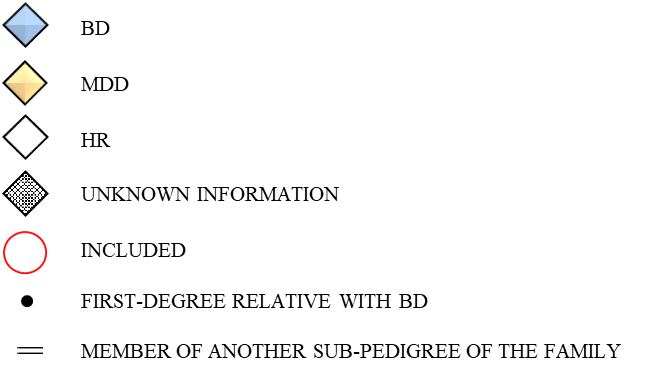


**Figure S6**. Pedigree of family 2, including twenty-three participants. Family 2 was divided in 8 subpedigrees.


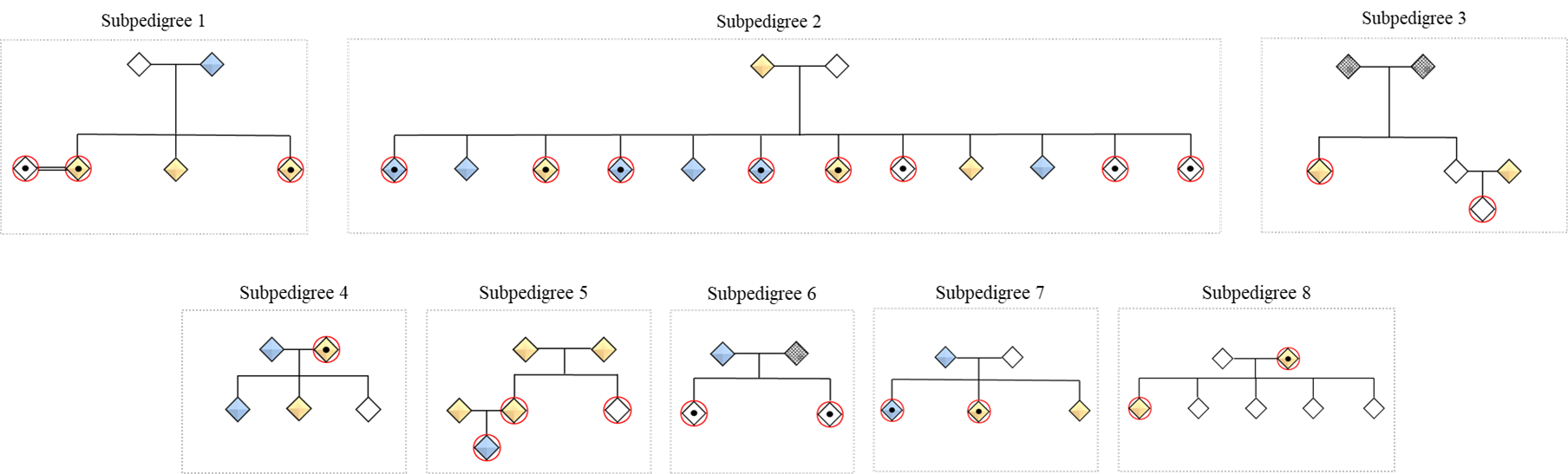

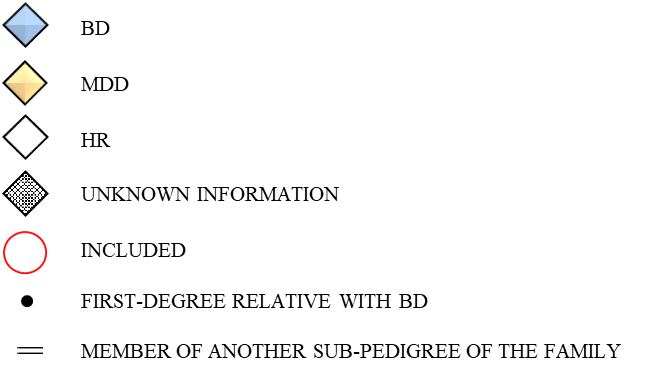

Supplement: Supplementary file 1 — Table S1. Information on individuals recruited from each family. Total number of pedigrees and number of individuals per pedigree. Table S2. Between‐group comparison of response inhibition, delay aversion, decision‐making, and risk behavior in alternative approach. Table S3. Between‐group comparison of response inhibition, delay aversion, decision‐making, and risk behavior in alternative approach with sex and age adjustment. Table S4. Between‐group comparison of response inhibition, delay aversion, decision‐making, and risk behavior with GEE models additionally adjusting for age and sex. Figure S1. Stop Signal Task comparison of subgroups. Figure S2. Delay aversion comparison of subgroups. Figure S3. Quality of decision‐making comparison of subgroups. Figure S4. Risk‐taking comparison of subgroups. Figure S5. Pedigree of family 1, including 24 participants. Family 1 was divided in subpedigrees 1–8. Figure S6. Pedigree of family 2, including 23 participants. Family 2 was divided in 8 subpedigrees. [file BRB3-14-e3337-s001.docx]
